# 2-Meth­oxy­imino-2-{2-[(2-methyl­phen­oxy)meth­yl]phen­yl}ethanol

**DOI:** 10.1107/S160053681203499X

**Published:** 2012-08-11

**Authors:** Rajni Kant, Vivek K. Gupta, Kamini Kapoor, Chetan S. Shripanavar, Kaushik Banerjee

**Affiliations:** a X-ray Crystallography Laboratory, Post-Graduate Department of Physics & Electronics, University of Jammu, Jammu Tawi 180 006, India; b National Research Centre for Grapes, Pune 412 307, India

## Abstract

In the title compound, C_17_H_19_NO_3_, the dihedral angle between the benzene rings is 68.0 (1)°. The C—O—C—C torsion angle of the atoms joining these rings is 179.7 (2)°. The atoms of the methanol group were refined as disordered over two sets of sites with fixed occupancies of 0.86 and 0.14. The H atoms of the hy­droxy group in the major component are disordered over a further two sets of sites with equal occupancies. This is a necessary arrangement to allow for hydrogen bonding without unrealistic H⋯H contacts. In the crystal, O—H⋯N and O—H⋯O hydrogen bonds connect mol­ecules into chains along [001].

## Related literature

The title compound was derived from kresoxim-methyl. For the biological activity of kresoxim-methyl, see: Anke *et al.* (1977 ▶); Clinton *et al.* (2011 ▶); Balba (2007 ▶); Sudisha *et al.* (2005 ▶). For related structures, see: Chopra *et al.* (2004 ▶); Kant *et al.* (2012*a* ▶,*b* ▶).
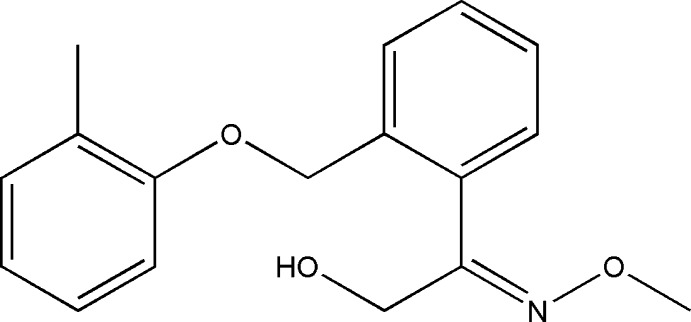


## Experimental

### 

#### Crystal data

C_17_H_19_NO_3_
*M* *_r_* = 285.33
Monoclinic, 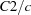

*a* = 21.0394 (14) Å
*b* = 20.4128 (10) Å
*c* = 7.6711 (5) Å
β = 105.729 (6)°
*V* = 3171.2 (3) Å^3^
*Z* = 8
Mo *K*α radiation
μ = 0.08 mm^−1^
*T* = 293 K
0.3 × 0.2 × 0.1 mm

#### Data collection

Oxford Diffraction Xcalibur Sapphire3 diffractometer
Absorption correction: multi-scan (*CrysAlis PRO*; Oxford Diffraction, 2010 ▶) *T*_min_ = 0.790, *T*_max_ = 1.000
11340 measured reflections
2788 independent reflections
1497 reflections with *I* > 2σ(*I*)
*R*_int_ = 0.052

#### Refinement

*R*[*F* ^2^ > 2σ(*F* ^2^)] = 0.066
*wR*(*F* ^2^) = 0.212
*S* = 1.08
2788 reflections
204 parameters
2 restraints
H-atom parameters constrained
Δρ_max_ = 0.39 e Å^−3^
Δρ_min_ = −0.22 e Å^−3^

### 

Data collection: *CrysAlis PRO* (Oxford Diffraction, 2010 ▶); cell refinement: *CrysAlis PRO*; data reduction: *CrysAlis PRO*; program(s) used to solve structure: *SHELXS97* (Sheldrick, 2008 ▶); program(s) used to refine structure: *SHELXL97* (Sheldrick, 2008 ▶); molecular graphics: *ORTEP-3* (Farrugia, 1997 ▶) and *SHELXTL* (Sheldrick, 2008 ▶); software used to prepare material for publication: *PLATON* (Spek, 2009 ▶).

## Supplementary Material

Crystal structure: contains datablock(s) I, global. DOI: 10.1107/S160053681203499X/lh5508sup1.cif

Structure factors: contains datablock(s) I. DOI: 10.1107/S160053681203499X/lh5508Isup2.hkl

Supplementary material file. DOI: 10.1107/S160053681203499X/lh5508Isup3.cml

Additional supplementary materials:  crystallographic information; 3D view; checkCIF report

## Figures and Tables

**Table 1:** Hydrogen-bond geometry (Å, °)

*D*—H⋯*A*	*D*—H	H⋯*A*	*D*⋯*A*	*D*—H⋯*A*
O11*A*—H11*Y*⋯O11*A* ^i^	0.84	1.77	2.614 (8)	178
O11*A*—H11*Z*⋯O11*A* ^ii^	0.84	2.11	2.950 (14)	178
O11*B*—H11*X*⋯N3^iii^	0.84	2.21	3.046 (18)	177

Symmetry codes: (i) 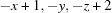; (ii) 
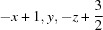
; (iii) 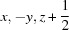.

**Fig. 1.**
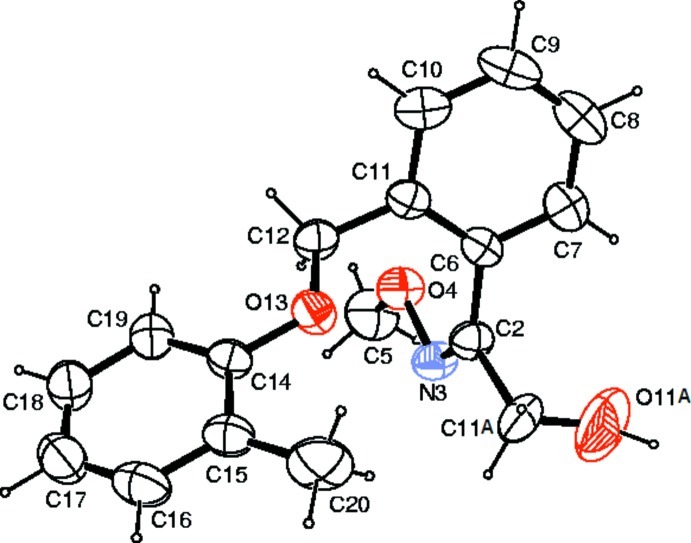
The molecular structure of the title compound with ellipsoids drawn at the 40% probability level.

**Fig. 2.**
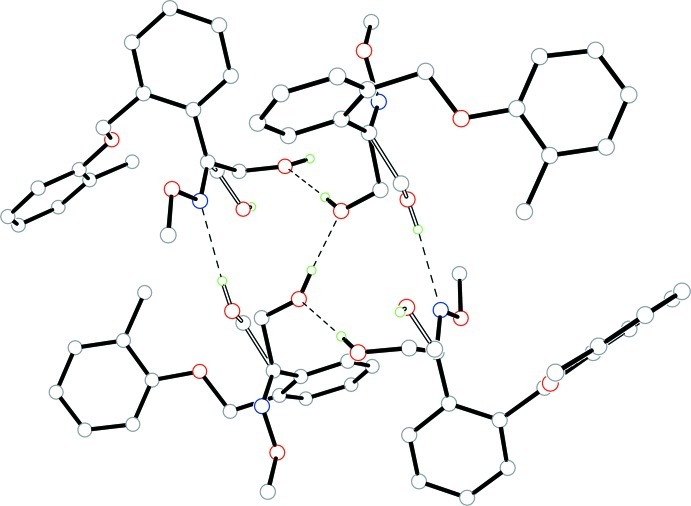
Part of the crystal structure showing the hydrogen bonds along [001] as dashed lines. For the other disorder component in the O—H···O hydrogen bonds, the acceptors become donors and vice-versa.

## supplementary crystallographic information

### Comment

Kresoxim-methyl is a widely used agricultural fungicide of the strobilurin group (Anke *et al.*, 1977; Clinton *et al.*, 2011; Balba, 2007). It is a broad-spectrum systemic compound with novel mode of action (Sudisha *et al.*, 2005). While exploring its fate in the environment, we have derived a new compound by the process of reduction. This may contribute to the understanding of the metabolic and environmental fate of this compound. The crystal structure of the title compound (I) is presented herein.

In (I)(Fig. 1), all bond lengths and angles are normal and correspond to those observed in the related structures (Chopra *et al.*, 2004; Kant *et al.*, 2012*a*,*b*). The dihedral angle between the two benzene rings is 68.0 (1)°. The C—O—C—C torsion angle of the atoms joining these rings is 179.7 (2) °. The atoms of the methanol group were refined as disordered over two sets of sites with fixed occupancies of 0.86 and 0.14. The H atoms of the hydroxy group in the major component are disordered over a further two sets of sites with equal occupancies. This is a necessary arrangement to allow for hydrogen bonding without unrealistic H···H contacts. The O—H···O hydrogen bond motif of one the O—H disorder components is shown in Fig. 2. For the other disorder component in the O—H···O hydrogen bonds, the acceptors become donors and *vice*-*versa*. In the crystal, O—H···N and O—H···O hydrogen bonds connect molecules to form chains along [001].

### Experimental

Finely powdered sodium borohydride (6 eq., 0.06 mol) was suspended in tetrahydrofuran in presence of kresoxim-methyl (3.13 g m, 0.01 mol) under reflux (343 K) with stirring for 1 h. Then methanol (8 ml) was slowly added drop wise. Stirring and refluxing were maintained until the reaction was completed as monitored by TLC. After the end of the reaction, the reaction mixture was cooled to room temperature and quenched with a saturated solution of ammonium chloride (15 ml) for further period of 1.5 h. The product was separated by extraction with ethyl acetate (2x25 ml). The organic extracts were combined and dried over sodium sulfate and concentrated under low pressure to yield the final product. The synthesized compound was dissolved in methanol and subjected to slow evaporation to produce colourless crystals.

### Refinement

All H atoms were positioned geometrically and were treated as riding on their parent atoms, with O—H distance of 0.84 Å and C—H distances of 0.93–0.97 Å and with *U*_iso_(H) = 1.2*U*_eq_(C) or 1.5*U*_eq_(methyl C,*O*). The disordered H atoms of the hydroxy group were placed in calculated positions which gave the most sensible hydrogen bonds.

### Crystal data

**Table:**

C_17_H_19_NO_3_	*F*(000) = 1216
*M**_r_* = 285.33	*D*_x_ = 1.195 Mg m^−^^3^
Monoclinic, *C*2/*c*	Mo *K*α radiation, λ = 0.71073 Å
Hall symbol: -C 2yc	Cell parameters from 2909 reflections
*a* = 21.0394 (14) Å	θ = 3.6–29.0°
*b* = 20.4128 (10) Å	µ = 0.08 mm^−^^1^
*c* = 7.6711 (5) Å	*T* = 293 K
β = 105.729 (6)°	Block, colourless
*V* = 3171.2 (3) Å^3^	0.3 × 0.2 × 0.1 mm
*Z* = 8	

### Data collection

**Table:**

Oxford Diffraction Xcalibur Sapphire3 diffractometer	2788 independent reflections
Radiation source: fine-focus sealed tube	1497 reflections with *I* > 2σ(*I*)
Graphite monochromator	*R*_int_ = 0.052
Detector resolution: 16.1049 pixels mm^-1^	θ_max_ = 25.0°, θ_min_ = 3.6°
ω scans	*h* = −23→24
Absorption correction: multi-scan (*CrysAlis PRO*; Oxford Diffraction, 2010)	*k* = −23→24
*T*_min_ = 0.790, *T*_max_ = 1.000	*l* = −9→9
11340 measured reflections	

### Refinement

**Table:**

Refinement on *F*^2^	Primary atom site location: structure-invariant direct methods
Least-squares matrix: full	Secondary atom site location: difference Fourier map
*R*[*F*^2^ > 2σ(*F*^2^)] = 0.066	Hydrogen site location: inferred from neighbouring sites
*wR*(*F*^2^) = 0.212	H-atom parameters constrained
*S* = 1.08	*w* = 1/[σ^2^(*F*_o_^2^) + (0.0921*P*)^2^] where *P* = (*F*_o_^2^ + 2*F*_c_^2^)/3
2788 reflections	(Δ/σ)_max_ = 0.002
204 parameters	Δρ_max_ = 0.39 e Å^−^^3^
2 restraints	Δρ_min_ = −0.22 e Å^−^^3^

### Special details

**Table:**

Experimental. *CrysAlis PRO*, Oxford Diffraction Ltd., Version 1.171.34.40 (release 27–08-2010 CrysAlis171. NET) (compiled Aug 27 2010,11:50:40) Empirical absorption correction using spherical harmonics, implemented in SCALE3 ABSPACK scaling algorithm.
Geometry. All e.s.d.'s (except the e.s.d. in the dihedral angle between two l.s. planes) are estimated using the full covariance matrix. The cell e.s.d.'s are taken into account individually in the estimation of e.s.d.'s in distances, angles and torsion angles; correlations between e.s.d.'s in cell parameters are only used when they are defined by crystal symmetry. An approximate (isotropic) treatment of cell e.s.d.'s is used for estimating e.s.d.'s involving l.s. planes.
Refinement. Refinement of *F*^2^ against ALL reflections. The weighted *R*-factor *wR* and goodness of fit *S* are based on *F*^2^, conventional *R*-factors *R* are based on *F*, with *F* set to zero for negative *F*^2^. The threshold expression of *F*^2^ > σ(*F*^2^) is used only for calculating *R*-factors(gt) *etc*. and is not relevant to the choice of reflections for refinement. *R*-factors based on *F*^2^ are statistically about twice as large as those based on *F*, and *R*- factors based on ALL data will be even larger.

### Fractional atomic coordinates and isotropic or equivalent isotropic displacement parameters (Å^2^)

**Table:**

	*x*	*y*	*z*	*U*_iso_*/*U*_eq_	Occ. (<1)
O4	0.36797 (11)	0.16370 (9)	0.4565 (3)	0.0749 (7)	
O13	0.24680 (10)	0.14485 (9)	0.7894 (3)	0.0689 (7)	
N3	0.37326 (13)	0.10321 (12)	0.5462 (4)	0.0709 (8)	
C2	0.38302 (15)	0.11036 (14)	0.7167 (4)	0.0638 (8)	
C5	0.3638 (2)	0.1523 (2)	0.2722 (5)	0.1041 (13)	
H5A	0.3630	0.1934	0.2110	0.156*	
H5B	0.3242	0.1283	0.2174	0.156*	
H5C	0.4014	0.1273	0.2629	0.156*	
C6	0.39179 (15)	0.17396 (14)	0.8143 (3)	0.0544 (8)	
C7	0.45455 (16)	0.19128 (17)	0.9162 (4)	0.0736 (9)	
H7A	0.4898	0.1630	0.9228	0.088*	
C8	0.4652 (2)	0.2500 (2)	1.0079 (5)	0.0912 (12)	
H8A	0.5077	0.2616	1.0736	0.109*	
C9	0.4141 (3)	0.2911 (2)	1.0029 (5)	0.0943 (12)	
H9A	0.4216	0.3307	1.0653	0.113*	
C10	0.3504 (2)	0.27400 (16)	0.9042 (5)	0.0785 (10)	
H10A	0.3155	0.3022	0.9023	0.094*	
C11	0.33831 (16)	0.21524 (14)	0.8083 (4)	0.0577 (8)	
C12	0.27043 (14)	0.19845 (14)	0.7035 (4)	0.0635 (9)	
H12A	0.2418	0.2361	0.6974	0.076*	
H12B	0.2701	0.1864	0.5810	0.076*	
C14	0.18363 (16)	0.12314 (14)	0.7087 (4)	0.0596 (8)	
C15	0.16296 (18)	0.06974 (15)	0.7930 (5)	0.0722 (9)	
C16	0.0989 (2)	0.04775 (18)	0.7189 (6)	0.0901 (12)	
H16A	0.0832	0.0129	0.7735	0.108*	
C17	0.0578 (2)	0.0762 (2)	0.5666 (7)	0.0991 (13)	
H17A	0.0152	0.0604	0.5195	0.119*	
C18	0.07975 (19)	0.1270 (2)	0.4858 (5)	0.0902 (12)	
H18A	0.0523	0.1457	0.3820	0.108*	
C19	0.14248 (17)	0.15098 (15)	0.5562 (5)	0.0737 (9)	
H19A	0.1572	0.1862	0.5006	0.088*	
C20	0.2088 (2)	0.03868 (18)	0.9558 (5)	0.1147 (15)	
H20A	0.2484	0.0250	0.9269	0.172*	
H20B	0.1877	0.0013	0.9919	0.172*	
H20C	0.2197	0.0698	1.0532	0.172*	
O11A	0.4567 (3)	0.0312 (2)	0.8725 (8)	0.253 (3)	0.86
H11Y	0.4837	0.0106	0.9553	0.380*	0.86
H11Z	0.4806	0.0312	0.8007	0.380*	0.86
C11A	0.3946 (2)	0.0467 (2)	0.8269 (7)	0.0952 (17)	0.86
H11A	0.3796	0.0522	0.9351	0.114*	0.43
H11B	0.3691	0.0116	0.7556	0.114*	0.43
O11B	0.3776 (10)	−0.0022 (7)	0.757 (2)	0.107 (7)	0.14
H11X	0.3746	−0.0298	0.8361	0.161*	0.14
C11B	0.3637 (19)	0.0532 (6)	0.822 (4)	0.0952 (17)	0.14
H11C	0.3875	0.0566	0.9491	0.114*	0.14
H11D	0.3168	0.0552	0.8123	0.114*	0.14

### Atomic displacement parameters (Å^2^)

**Table:**

	*U*^11^	*U*^22^	*U*^33^	*U*^12^	*U*^13^	*U*^23^
O4	0.1028 (18)	0.0737 (14)	0.0500 (13)	−0.0026 (12)	0.0234 (11)	0.0048 (10)
O13	0.0712 (15)	0.0688 (13)	0.0615 (13)	−0.0153 (11)	0.0090 (11)	0.0183 (10)
N3	0.091 (2)	0.0592 (15)	0.0623 (18)	0.0014 (13)	0.0212 (14)	−0.0015 (13)
C2	0.080 (2)	0.0564 (18)	0.0553 (19)	−0.0021 (16)	0.0193 (15)	0.0060 (15)
C5	0.141 (4)	0.120 (3)	0.050 (2)	−0.007 (3)	0.023 (2)	−0.008 (2)
C6	0.067 (2)	0.0615 (18)	0.0362 (16)	−0.0124 (16)	0.0163 (14)	0.0013 (12)
C7	0.074 (2)	0.095 (2)	0.054 (2)	−0.0094 (19)	0.0206 (17)	−0.0034 (18)
C8	0.088 (3)	0.118 (3)	0.070 (2)	−0.043 (3)	0.026 (2)	−0.019 (2)
C9	0.128 (4)	0.081 (3)	0.083 (3)	−0.043 (3)	0.045 (3)	−0.030 (2)
C10	0.110 (3)	0.063 (2)	0.075 (2)	−0.004 (2)	0.046 (2)	−0.0010 (17)
C11	0.071 (2)	0.0549 (17)	0.0504 (17)	−0.0103 (17)	0.0220 (15)	0.0061 (13)
C12	0.073 (2)	0.0567 (18)	0.0632 (19)	−0.0037 (15)	0.0227 (16)	0.0184 (15)
C14	0.067 (2)	0.0572 (18)	0.0570 (19)	−0.0061 (16)	0.0201 (16)	−0.0041 (14)
C15	0.082 (3)	0.0623 (19)	0.078 (2)	−0.0110 (19)	0.0319 (19)	−0.0012 (17)
C16	0.094 (3)	0.073 (2)	0.115 (3)	−0.025 (2)	0.050 (3)	−0.016 (2)
C17	0.070 (3)	0.096 (3)	0.130 (4)	−0.011 (2)	0.026 (3)	−0.035 (3)
C18	0.071 (3)	0.092 (3)	0.101 (3)	0.004 (2)	0.011 (2)	−0.012 (2)
C19	0.068 (2)	0.075 (2)	0.076 (2)	0.0006 (19)	0.0161 (18)	0.0016 (17)
C20	0.145 (4)	0.092 (3)	0.110 (3)	−0.029 (2)	0.038 (3)	0.036 (2)
O11A	0.166 (5)	0.175 (4)	0.355 (9)	0.019 (4)	−0.040 (4)	0.159 (5)
C11A	0.089 (5)	0.075 (3)	0.113 (4)	0.026 (3)	0.012 (3)	0.024 (3)
O11B	0.20 (2)	0.031 (8)	0.083 (12)	0.001 (11)	0.026 (12)	−0.011 (8)
C11B	0.089 (5)	0.075 (3)	0.113 (4)	0.026 (3)	0.012 (3)	0.024 (3)

### Geometric parameters (Å, º)

**Table:**

O4—N3	1.403 (3)	C14—C19	1.375 (4)
O4—C5	1.412 (4)	C14—C15	1.396 (4)
O13—C14	1.378 (3)	C15—C16	1.388 (5)
O13—C12	1.434 (3)	C15—C20	1.497 (5)
N3—C2	1.277 (3)	C16—C17	1.379 (5)
C2—C6	1.485 (4)	C16—H16A	0.9300
C2—C11A	1.533 (5)	C17—C18	1.352 (5)
C2—C11B	1.535 (7)	C17—H17A	0.9300
C5—H5A	0.9600	C18—C19	1.374 (4)
C5—H5B	0.9600	C18—H18A	0.9300
C5—H5C	0.9600	C19—H19A	0.9300
C6—C7	1.386 (4)	C20—H20A	0.9600
C6—C11	1.396 (4)	C20—H20B	0.9600
C7—C8	1.378 (5)	C20—H20C	0.9600
C7—H7A	0.9300	O11A—C11A	1.298 (6)
C8—C9	1.355 (5)	O11A—H11Y	0.8400
C8—H8A	0.9300	O11A—H11Z	0.8399
C9—C10	1.394 (5)	C11A—H11A	0.9700
C9—H9A	0.9300	C11A—H11B	0.9700
C10—C11	1.394 (4)	O11B—C11B	1.300 (8)
C10—H10A	0.9300	O11B—H11X	0.8400
C11—C12	1.477 (4)	C11B—H11C	0.9700
C12—H12A	0.9700	C11B—H11D	0.9700
C12—H12B	0.9700		
			
N3—O4—C5	108.7 (2)	C19—C14—C15	120.9 (3)
C14—O13—C12	116.8 (2)	O13—C14—C15	115.2 (3)
C2—N3—O4	111.8 (2)	C16—C15—C14	116.9 (3)
N3—C2—C6	125.5 (3)	C16—C15—C20	122.6 (3)
N3—C2—C11A	115.2 (3)	C14—C15—C20	120.4 (3)
C6—C2—C11A	118.9 (3)	C17—C16—C15	121.8 (4)
N3—C2—C11B	117.4 (12)	C17—C16—H16A	119.1
C6—C2—C11B	114.4 (9)	C15—C16—H16A	119.1
O4—C5—H5A	109.5	C18—C17—C16	119.9 (4)
O4—C5—H5B	109.5	C18—C17—H17A	120.1
H5A—C5—H5B	109.5	C16—C17—H17A	120.1
O4—C5—H5C	109.5	C17—C18—C19	120.3 (4)
H5A—C5—H5C	109.5	C17—C18—H18A	119.9
H5B—C5—H5C	109.5	C19—C18—H18A	119.9
C7—C6—C11	120.1 (3)	C18—C19—C14	120.2 (3)
C7—C6—C2	118.5 (3)	C18—C19—H19A	119.9
C11—C6—C2	121.5 (3)	C14—C19—H19A	119.9
C8—C7—C6	120.5 (3)	C15—C20—H20A	109.5
C8—C7—H7A	119.7	C15—C20—H20B	109.5
C6—C7—H7A	119.7	H20A—C20—H20B	109.5
C9—C8—C7	120.4 (4)	C15—C20—H20C	109.5
C9—C8—H8A	119.8	H20A—C20—H20C	109.5
C7—C8—H8A	119.8	H20B—C20—H20C	109.5
C8—C9—C10	119.9 (3)	C11A—O11A—H11Y	138.5
C8—C9—H9A	120.0	C11A—O11A—H11Z	124.1
C10—C9—H9A	120.0	H11Y—O11A—H11Z	95.5
C9—C10—C11	120.9 (3)	O11A—C11A—C2	110.6 (4)
C9—C10—H10A	119.6	O11A—C11A—H11A	109.5
C11—C10—H10A	119.6	C2—C11A—H11A	109.5
C10—C11—C6	118.1 (3)	O11A—C11A—H11B	109.5
C10—C11—C12	119.9 (3)	C2—C11A—H11B	109.5
C6—C11—C12	122.0 (3)	H11A—C11A—H11B	108.1
O13—C12—C11	109.4 (2)	C11B—O11B—H11X	104.0
O13—C12—H12A	109.8	O11B—C11B—C2	109.9 (12)
C11—C12—H12A	109.8	O11B—C11B—H11C	109.7
O13—C12—H12B	109.8	C2—C11B—H11C	109.7
C11—C12—H12B	109.8	O11B—C11B—H11D	109.7
H12A—C12—H12B	108.2	C2—C11B—H11D	109.7
C19—C14—O13	123.9 (3)	H11C—C11B—H11D	108.2
			
C5—O4—N3—C2	174.3 (3)	C10—C11—C12—O13	−110.4 (3)
O4—N3—C2—C6	−3.3 (4)	C6—C11—C12—O13	69.9 (3)
O4—N3—C2—C11A	−175.6 (3)	C12—O13—C14—C19	−1.9 (4)
O4—N3—C2—C11B	157.0 (14)	C12—O13—C14—C15	178.2 (2)
N3—C2—C6—C7	−105.8 (4)	C19—C14—C15—C16	−1.8 (5)
C11A—C2—C6—C7	66.3 (4)	O13—C14—C15—C16	178.0 (3)
C11B—C2—C6—C7	93.5 (16)	C19—C14—C15—C20	178.3 (3)
N3—C2—C6—C11	75.6 (4)	O13—C14—C15—C20	−1.8 (4)
C11A—C2—C6—C11	−112.3 (3)	C14—C15—C16—C17	1.5 (5)
C11B—C2—C6—C11	−85.1 (16)	C20—C15—C16—C17	−178.6 (4)
C11—C6—C7—C8	−1.9 (4)	C15—C16—C17—C18	−0.2 (6)
C2—C6—C7—C8	179.5 (3)	C16—C17—C18—C19	−0.9 (6)
C6—C7—C8—C9	1.5 (5)	C17—C18—C19—C14	0.5 (5)
C7—C8—C9—C10	−0.1 (5)	O13—C14—C19—C18	−179.0 (3)
C8—C9—C10—C11	−0.7 (5)	C15—C14—C19—C18	0.9 (5)
C9—C10—C11—C6	0.2 (4)	N3—C2—C11A—O11A	87.9 (5)
C9—C10—C11—C12	−179.5 (3)	C6—C2—C11A—O11A	−85.0 (5)
C7—C6—C11—C10	1.1 (4)	C11B—C2—C11A—O11A	−171 (3)
C2—C6—C11—C10	179.6 (3)	N3—C2—C11B—O11B	38 (3)
C7—C6—C11—C12	−179.2 (2)	C6—C2—C11B—O11B	−160 (2)
C2—C6—C11—C12	−0.6 (4)	C11A—C2—C11B—O11B	−53.1 (15)
C14—O13—C12—C11	179.7 (2)		

### Hydrogen-bond geometry (Å, º)

**Table:**

*D*—H···*A*	*D*—H	H···*A*	*D*···*A*	*D*—H···*A*
O11*A*—H11*Y*···O11*A*^i^	0.84	1.77	2.614 (8)	178
O11*A*—H11*Z*···O11*A*^ii^	0.84	2.11	2.950 (14)	178
O11*B*—H11*X*···N3^iii^	0.84	2.21	3.046 (18)	177

Symmetry codes: (i) −*x*+1, −*y*, −*z*+2; (ii) −*x*+1, *y*, −*z*+3/2; (iii) *x*, −*y*, *z*+1/2.
